# Transcriptional response of *Asarum heterotropoides* Fr. Schmidt var. *mandshuricum* (Maxim.) Kitag. leaves grown under full and partial daylight conditions

**DOI:** 10.1186/s12864-020-07266-7

**Published:** 2021-01-06

**Authors:** Zhiqing Wang, Haiqin Ma, Min Zhang, Ziqing Wang, Yixin Tian, Wei Li, Yingping Wang

**Affiliations:** 1 Laboratory of Cultivation and Breeding of Medicinal Plants, National Administration of Traditional Chinese Medicine, College of Chinese Medicinal Materials, Jilin Agricultural University, Changchun, 130118 Jilin China; 2 Institute of Special Wild Economic Animals and Plants, Chinese Academy of Agriculture Sciences, Changchun, 130112 Jilin China; 3 State & Local Joint Engineering Research Center of Ginseng Breeding and Application, College of Chinese Medicinal Materials, Jilin Agricultural University, Changchun, 130118 Jilin China

**Keywords:** Hormone signaling, Herbal plant, Photosynthesis, Sciophyte, Transcriptome, Volatile oil, Bioactive component

## Abstract

**Background:**

*Asarum heterotropides* Fr. Schmidt var. *mandshuricum* (Maxim.) Kitag. is an important medicinal and industrial plant, which is used in the treatment of various diseases. The main bioactive ingredient is the volatile oil having more than 82 identified components of which methyleugenol, safrole, myristicin, and toluene account for about 70% of the total volume. As a sciophyte plant, the amount of light it absorbs through leaves is an important factor for growth and metabolism.

**Results:**

We grew *Asarum* plants under full, 50, 28, and 12% sunlight conditions to investigate the effect of different light irradiances on the four major volatile oil components. We employed de novo transcriptome sequencing to understand the transcriptional behavior of *Asarum* leaves regarding the biosynthetic pathways of the four volatile oil components, photosynthesis and biomass accumulation, and hormone signaling. Our results demonstrated that the increasing light conditions promoted higher percent of the four components. Under full sunlight conditions, cinnamyl alcohol dehydrogenase and cytochrome p450719As were upregulated and led the increased methyleugenol, safrole, and myristicin. The transcriptomic data also showed that *Asarum* leaves, under full sunlight conditions, adjust their photosynthesis-antenna proteins as a photoprotective response with the help of carotenoids. Plant hormone-signaling related genes were also differentially expressed between full sunlight and low light conditions.

**Conclusions:**

High light induces accumulation of major bioactive ingredients *A. heterotropides* volatile oil and this is ascribed to upregulation of key genes such as cinnamyl alcohol dehydrogenase and cytochrome p450719As. The transcriptome data presented here lays the foundation of further understanding of light responses in sciophytes and provides guidance for increasing bioactive molecules in *Asarum*.

**Supplementary Information:**

The online version contains supplementary material available at 10.1186/s12864-020-07266-7.

## Background

*Asarum heterotropoides* Fr. Schmidt var. *mandshuricum* (Maxim.) Kitag., a perennial herb endemic to China, has been exploited as a traditional medicinal herb due to its anti-inflammatory, anti-bacterial, anti-pyretic, anticancer, fungistatic and analgesic properties [1, 2]. This species has a wide geographical distribution and grows in shady habitats and mountainous wetlands. The main producing areas are Jilin, Liaoning, and Heilongjiang in China [3]. Previous studies have documented that the main bioactive ingredient is the essential oil for which more than 82 components have been identified [4, 5]. As a sciophyte, different studies have demonstrated that growing in different solar irradiance levels affects the leaf mass to per unit area of the plant, chlorophyll content, and net photosynthetic rate [6]. Two recent studies demonstrated that light intensities affect photosynthesis and chlorophyll content but the content of *Asarum* volatile oil did not change among different groups [6, 7]. However, the study by Wang et al., [7] used GC-MS to determine the composition of the oil and reported variation in oil composition under different light treatments suggesting that light treatments somehow affect the regulation of the pathways involved in volatile oil biosynthesis. The main components of *Asarum* essential oil used in the pharmaceutical industry are phenylpropane compounds including methyleugenol, safrole, myristicin, 1,3-benzodioxole, 4-methoxy-6- (2-propenyl)-, 3,5-dimethoxytoluene, 2-Hydroxy-4,5- methylenedioxypropiophenone, etc., these compounds account for about 70% of the total volatile oil content [4]. In addition to the above mentioned recent studies, a previous investigation reported that the content of major components was subjected to seasonal variation [8]. In other species, the effect of light intensities on growth and accumulation of essential oils and secondary metabolites has been established e.g. manipulation of the light affected the secondary metabolite contents in *Glycyrrhiza uralensis* Fisch [9]. Similarly, solar irradiance levels altered volatile oil contents in basil (*Ocimum basilicum* L.), *Myrtus communis* L., *Ocimum gratissimum*, damask rose (*Rosa damascena* Mill.), and other aromatic plants [10–13]. These contradicting reports suggest that a deeper understanding is a prerequisite for establishing an optimal irradiation protocol for *Asarum* growth, which can provide high yield of volatile oil and its major bioactive components for industrial scale volatile oils.

Methyleugenol is a common phenylpropanoid found in many medicinal plant species. It is derived from eugenol that is a product of phenylalanine through the reaction of cinnamic acid, ferulic acid, coniferyl alcohol, and coniferyl acetate. Methyleugenol is further converted into myristicin [14–17]. Several reports have identified and characterized this pathway-related genes e.g. coniferyl alcohol acyl transferase (CAAT) in apple fruit, eugenol synthase genes (EGS) in rose, Ocimum, and Gymnadenia, genes encoding O-methyltransferases (OMT) in loquat, cinnamyl alcohol dehydrogenase (CAD) in Arabidopsis and many other plant species [18–24]. Another important constituent of the volatile oil in *Asarum* is safrole. It has been suggested that it is possibly biosynthesized from eugenol through the formation of the methylenedioxy bridge and shares a common precursor coniferyl alcohol [25]. Apart from methyleugenol, myristicin, and safrole, toluene (3,5-dimethoxytoluene) is another major component of volatile oil in many aromatic medicinal plants including *Asarum* species. In roses, a number of OMT genes have been identified to convert orcinol to 3-methoxyl-5-hydroxytoluene, and then to toluene [26, 27]. Because, these four components are the main components in the *Asarum* volatile oil, it is important to understand their possible regulation under different light intensities.

The amount of solar radiation directly impacts on the photosynthetic characteristics of *Asarum* [6] and it is known that the biomass accumulation is associated with the rate of photosynthesis in plants. Therefore, it is essential to understand the regulation of genes involved in plant biomass accumulation and photosynthetic efficiency in *Asarum* together with the impact of irradiance on volatile oil components [28]. In plants, photosynthesis is a complex, multistep process involving electron transport chain, Calvin-Benson cycle, and subsequent steps involving assimilation, transport, and utilization of photoassimilates. These distinct yet overlapping processes require the product of hundreds of proteins and genes associated either with the nucleus or chloroplasts [29]. Similarly, biomass accumulation in plants is a complex process involving photosynthetic pathways, cell architecture, plant growth regulators, sugar transport and accumulation, metabolism, and regulation of transcription [30]. Since both processes involve a high number of genes and pathways, studying them in an individual genetic characterization project or working with a single pathway is not possible and demands large-scale transcriptome analyses. Recent developments in transcriptomics have enabled the understanding of complex pathways in medicinal plants including *Asarum* [25, 31–33]. The low light irradiance levels lead plant leaves to adapt in shade conditions through various mechanisms involving photosynthetic machinery, adjustment in cell growth, stomatal conductance, and hormone-signaling [13, 34–36]. Therefore, a transcriptome will enable the understanding of the differential changes in the expression of genes involved in these pathways.

Efforts have been made to optimize the extraction of volatile oil from this medicinally important plant [37]. The possible ways to increase the volatile oil production are 1) to understand the effect of different light intensities on the photosynthate and in turn on the volatile oil biosynthesis [38], 2) understand the volatile oil and its components’ biosynthesis-related pathways, and based on this knowledge, 3) develop high volatile oil yielding *A. heterotropoides* genotypes for large scale extraction. In this study, transcriptome sequencing of *Asarum* leaves grown under four different light treatments was performed to uncover the effect of light treatments on genes involved in pathways associated with the biosynthesis of methyleugenol, myristicin, safrole, and 3,5-dimethoxytoluene. Furthermore, we also studied the effect of light on the genes related to photosynthesis, which in turn influence biomass accumulation under different light irradiances. Additionally, we also explored the differential regulation of plant hormone signaling related genes.

## Results

### Effect of shade treatments on important volatile oil constituents

**Fig. 1:**
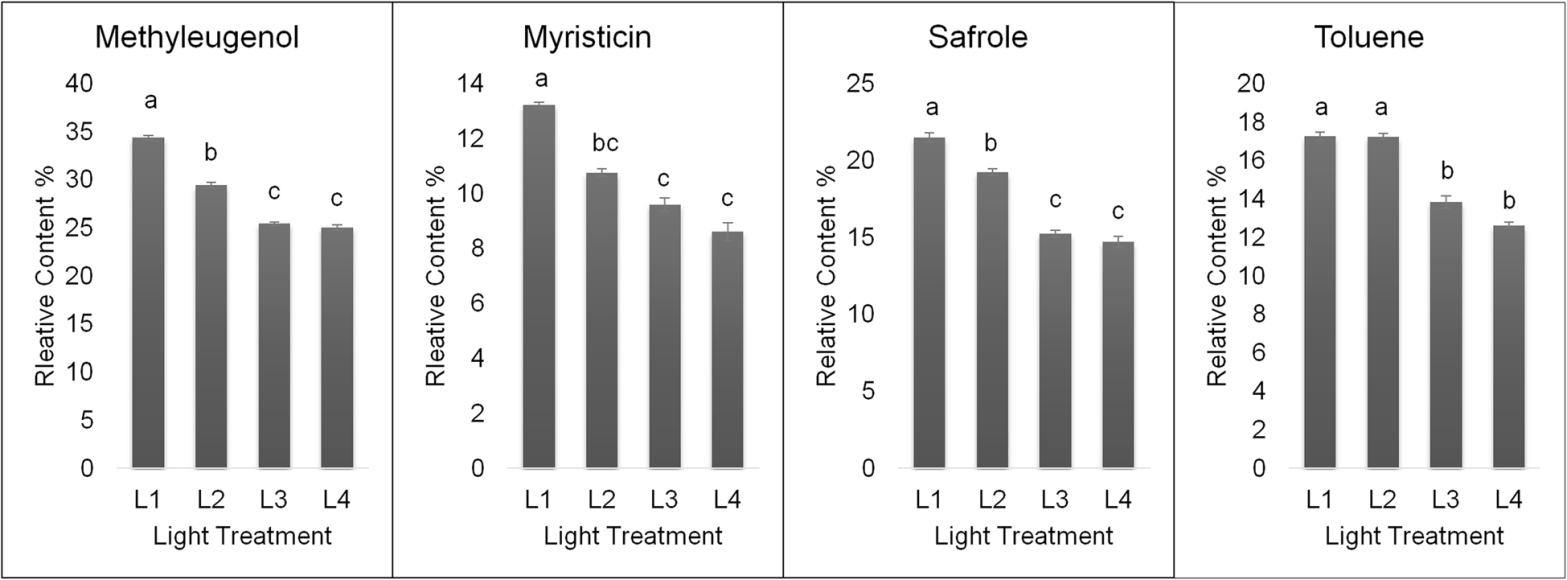
Effect of light treatments on methyleugenol, myristicin, safrole, and toluene percent in *Asarum* essential oil. Error bar represents SD from triplicate data. Means with the same letter are not significantly different from each other (*P* < 0.05). L1, L2, L3 and L4 represent full sunlight, 50% sunlight, 28% sunlight and 12% sunlight, respectively

**Fig. 2:**
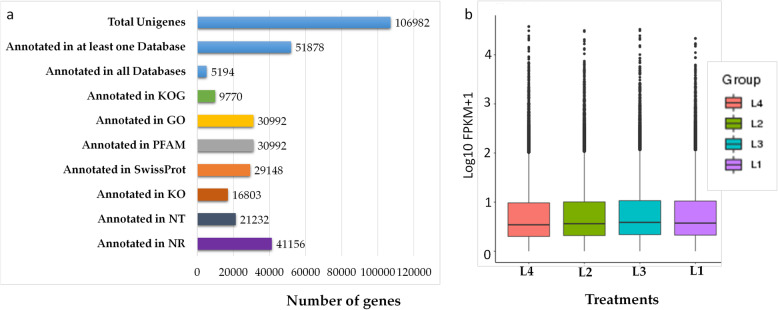
**a** Unigene database functional annotation statistics. **b** Distribution of gene expression in four light irradiation treatments in *Asarum* leaves. L1, L2, L3 and L4 represent full sunlight, 50% sunlight, 28% sunlight and 12% sunlight, respectively

*Asarum* plants were grown under four light irradiances including, full sunlight (L1), 50% sunlight (L2), 28% sunlight (L3), and 12% sunlight (L4). Because leaf is the plant organ, where light is directly absorbed and the main photosynthate is produced and processed, therefore, we focused on the essential oil changes and transcriptional responses as adopted in a previous studies [6]. The percent yield of four important *Asarum* volatile oil components under various light treatments are shown in Fig. 1. The oil component with the highest percentage among the four major volatile oil constituents was methyleugenol followed by safrole, toluene, and myristicin. Metyleugenol content was highest under full sunlight conditions and decreased with the decrease in the light intensity while it did not differ significantly between 28 and 12% light conditions. Myristicin showed an almost similar pattern where the highest content was recorded in full sunlight grown leaves and it decreased with the reduction in the light intensity. It decreased 9.75, 8.58, and 7.6 fold when treated with 50, 28, and 12% light, respectively. Safrole content also showed a reducing pattern with the decrease in the light intensity where the highest content was 21.4% in full sunlight conditions followed by 19.21, 15.26, and 14.70% when leaves were grown under 50, 28, and 12% sunlight conditions, respectively. The toluene percent content in full and 50% sunlight light grown leaves did not differ significantly while a further reduction in sunlight intensity (28% light) resulted in 12.8 fold decrease. Under low light conditions i.e. 28 and 12% light, the toluene content did not change significantly, however, a reducing trend was still noticeable (Fig. 2). Together these observations suggest that *Asarum* leaves grown under higher light conditions result in volatile oil higher enriched in the four compounds.

### Overview of Transcriptome analyses

The cDNA libraries constructed from light treated *A. heterotropoides* leaves were sequenced with Illumina HiseqTM high-throughput sequencing platform. After filtering low quality reads and adapter sequences, a total of 97.33 Gb clean data was obtained consisting of Illumina reads ranging from 41,987,144 to 65,408,518 million/sample (average 54,067,052) (Additional Table 1). Trinity assembly tool was used to de novo assemble the transcriptome. After data processing, 106,982 unigene sequences were included, and the N50 was 1375 bp long. The summary of the unigene sequence size ranges is shown in Additional Fig. 1.

**Fig. 3:**
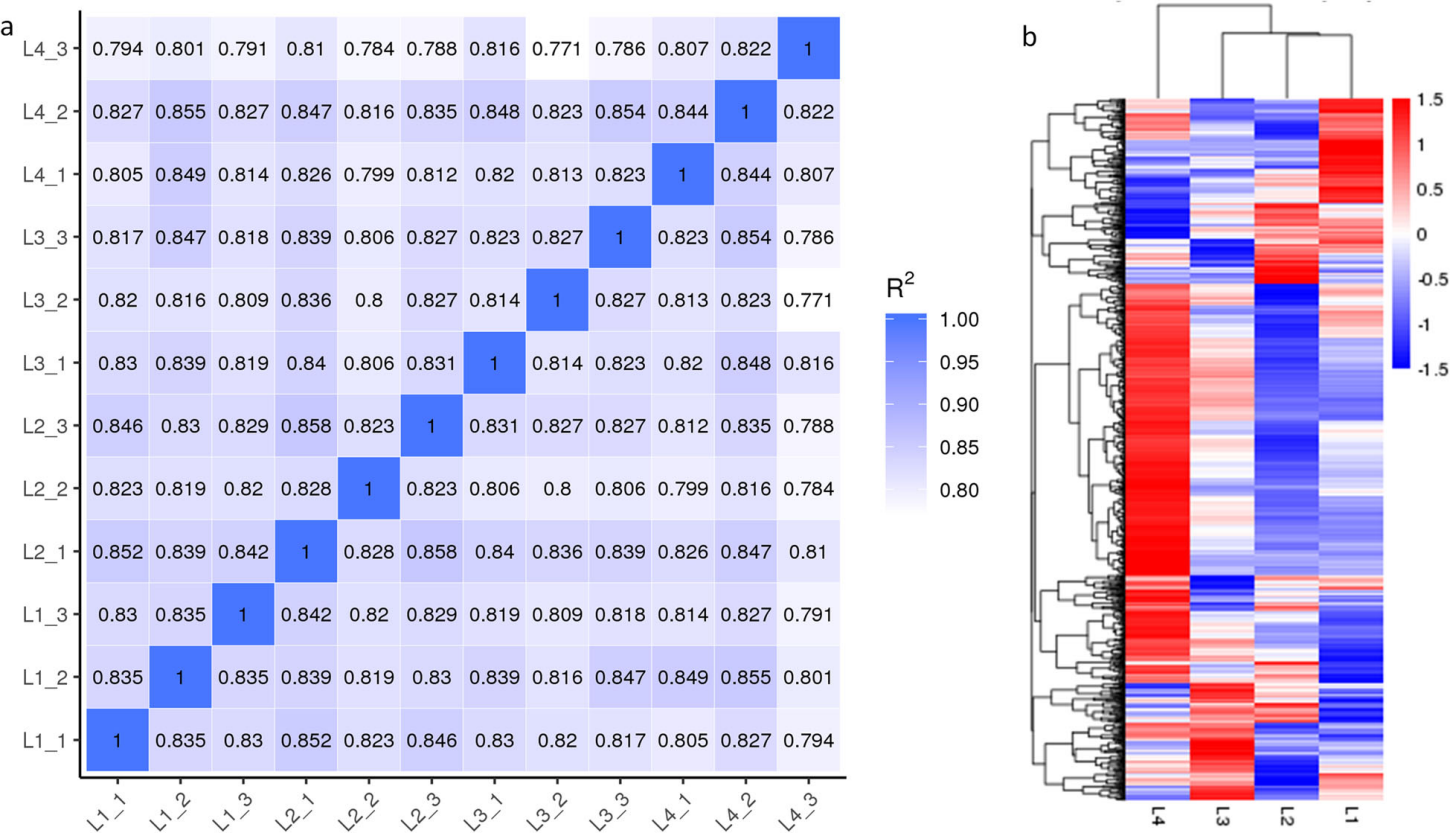
**a** Pearson correlations between replicates of four irradiation treatments in *Asarum* leaves, and **b)** differential gene heat map; the abscissa represents the sample name and hierarchical clustering results and the ordinate represents the differential genes and hierarchical clustering results. L1, L2, L3 and L4 represent full sunlight, 50% sunlight, 28% sunlight and 12% sunlight, respectively

Functional annotation of all unigenes as blasted the non-redundant (NR) (38.47%), Nucleotide (NT) (19.48%), Kyoto encyclopedia of genes and genomes (KEGG) (15.7%), Swiss-Port (27.24%), Pfam (28.96%), Gene Ontology (GO) (28.96%), Ortholog Groups (KOG) (9.13%) databases is presented in Fig. 2a; a total of 106,982 unigenes was annotated. The Fragments Per Kilobase of Transcript per Million Fragments Mapped (FPKM) gene expression levels in the four treatments are shown in Fig. 2b. Pearson correlations between replicates of the four irradiation treatments in *Asarum* leaves ranged from 0.771 to 0.858 (Fig. 3a). Differential expressed genes (DEGs) expressed under different treatment comparisons are shown in Fig. 3b and Additional Fig. 2. Graphical representation of the KEGG enrichment scatter plot of DEGs between different treatment comparisons is shown in Additional Fig. 3. We used the Richfactor, Q-value, and number of genes enriched in specific pathways to show the degree of KEGG enrichment. The KEGG pathway enrichment showed that the most common significantly enriched pathways under the tested conditions were phenylpropanoid pathway, plant hormone signaling-transduction, photosynthesis-antenna proteins, protein processing in the endoplasmic reticulum, and flavonoid metabolism (Additional Fig. 3).

### Transcriptomic response of *Asarum* leaves to light treatments

#### Differential regulation of volatile oil biosynthesis related genes

Previous studies have demonstrated that the volatile oil content is affected by light/shade conditions [6, 9, 13]. Therefore, we searched for the DEGs associated with volatile oil biosynthesis in our comparative transcriptome data between different light treated *Asarum* leaves. Between the full sunlight and 50% light conditions, a gene (*Cluster-24,085.27155*) was upregulated in *Asarum* leaves. This gene is annotated as CAD and the KEGG pathway mapping suggested its involvement in controlling the important steps in the formation of caffeyl-alchol and coniferyl alcohol, which are intermediates in the formation of methyleugenol (Additional Table 2). The upregulation suggested full sunlight conditions have a beneficial impact on the biosynthesis of methyleugenol through the upregulation of CAD gene. Two other genes involved in the same pathway i.e. the final steps of methyleugenol biosynthesis were also upregulated in full sunlight grown *Asarum* leaves as compared to 28% light conditions. One of the two genes was annotated as CAD (*Cluster-24,085.41570*) while the second gene was annotated as peroxidase 25-like (*Cluster-24,085.21345*) (Additional Table 3). This second gene is involved in the final step of lignin formation. Another gene (*Cluster-24,085.10149*) was upregulated in *Asarum* leaves grown in 50% light conditions when compared with 28% light conditions. This gene is involved in the formation of phenyl-ethylamine, which is an important intermediate of phenylpropanoid pathway (Additional Table 4; Table 1).

**Table 1:** DEGs related to Phenylpropanoid pathway associated with volatile oil biosynthesis in *Asarum* leaves grown under different light intensities. L1, L2, L3 and L4 represent full sunlight, 50% sunlight, 28% sunlight and 12% sunlight, respectively

Treatment	Gene ID	Log2 fold change	P value adjusted	Description
Phenylpropanoid Pathway				
L1 to L2	*Cluster-24,085.27155*	2.657	0.038336	Cinnamyl alcohol dehydrogenase 3
L1 to L3	*Cluster-24,085.21345*	2.2109	0.020262	Peroxidase 25-like
	*Cluster-24,085.41570*	2.5763	0.020982	Cinnamyl alcohol dehydrogenase
L1 to L4	*Cluster-24,085.23909*	2.2421	0.00372	trans-resveratrol O-methyltransferase
L2 to L3	*Cluster-24,085.10149*	2.7642	0.012531	aromatic-L-amino-acid decarboxylase-like

#### Differential regulation of photosynthesis and biomass accumulation related genes

As it is previously established that morphological, physiological, and biochemical changes occur in plants grown under sunlight versus shade conditions and the process of photosynthesis is affected [34], we searched for genes in our transcriptome that are associated directly or indirectly with photosynthesis. An important gene (*Cluster-24,085.63483*) implicated in porphyrin and chlorophyll metabolism pathway was upregulated (log2foldchange = 3.2) under full sunlight grown *Asarum* leaves as compared to 50% light conditions (Additional Table 2; Table 2). One photosynthesis-antenna protein related to light-harvesting complexes (*Cluster-24,085.41612*, Lhcb1) was downregulated in full sunlight conditions as compared to 50% light. This gene was also differentially expressed between full sunlight versus very low light intensity (12%) conditions (Additional Table 4). The downregulation under full sunlight conditions compared to both 50 and 12% light intensities suggests that under the influence of increased light intensity, *Asarum* optimizes their light-harvesting antenna. Because there are several photosynthesis-antenna proteins, we searched our DEGs for other antenna proteins and found the downregulation of one antenna protein (*Cluster-24,085.76692*, Lhcb2) in full sunlight versus 28% light conditions. Interestingly, five antenna proteins were downregulated in full versus 12% sunlight grown *Asarum* leaves (Table 3). These results further confirmed the fact that like other plants, *Asarum* leaves manipulate antenna proteins to control the light capture rate under natural full sunlight conditions.

**Table 2:** List of DEGs related to photosynthesis in *Asarum* leaves grown under different light intensities. L1, L2, L3 and L4 represent full sunlight, 50% sunlight, 28% sunlight and 12% sunlight, respectively

Treatment	Gene ID	Log2 fold change	Pvalue adjusted	Description
Carotenoid biosynthesis				
L1 to L2	*Cluster-9588.0*	4.514	0.034696	Beta-carotene 3-hydroxylase
L1 to L4	*Cluster-9588.0*	4.299	0.016687	Beta-carotene 3-hydroxylase
L2 to L4	*Cluster-24,085.17524*	−1.9954	0.001628	9-Cis-epoxycarotenoid dioxygenase
	*Cluster-24,085.21006*	−4.7706	3.46E-11	Abscisic acid 8′-hydroxylase
Photosynthesis - antenna proteins				
L1 to L2	*Cluster-24,085.41612*	−1.291	0.000785	Chlorophyll a-b binding protein
L1 to L3	*Cluster-24,085.76692*	−4.6487	0.023195	Chlorophyll a-b binding protein
L1 to L4	*Cluster-24,085.41606*	−1.7841	0.023063	Chlorophyll a-b binding protein
	*Cluster-24,085.76692*	−5.3381	0.022416	Chlorophyll a-b binding protein
	*Cluster-15,166.0*	−1.5438	0.023391	Chlorophyll a-b binding protein
	*Cluster-24,085.41612*	−1.9349	0.0097455	Chlorophyll a-b binding protein
	*Cluster-24,085.40718*	−1.8981	0.0008757	Chlorophyll a-b binding protein
Porphyrin and chlorophyll metabolism				
L1 to L2	*Cluster-24,085.63483*	3.2081	0.003801	UDP-glycosyltransferase 76F1-like

**Table 3:** List of DEGs related to biomass accumulation in *Asarum* leaves grown under different light intensities. L1, L2, L3 and L4 represent full sunlight, 50% sunlight, 28% sunlight and 12% sunlight, respectively

Treatment	Gene ID	Log2 fold change	Pvalue adjusted	Description
Ascorbate and aldarate metabolism				
L1 to L2	*Cluster-24,085.74455*	−1.6322	0.0091074	L-ascorbate oxidase
	*Cluster-24,085.63483*	3.2081	0.003801	UDP-glycosyltransferase 76F1-like
L1 to L4	*Cluster-24,085.74455*	−3.9989	2.04E-09	L-ascorbate oxidase
	*Cluster-24,085.74454*	−3.9441	3.30E-06	L-ascorbate oxidase
L2 to L4	*Cluster-24,085.74455*	−2.3647	0.001596	L-ascorbate oxidase
	*Cluster-24,085.50783*	−1.8271	0.016912	UDP-glycosyltransferase 76F1-like
L3to L4	*Cluster-24,085.74455*	−2.6414	0.0095753	L-ascorbate oxidase
Carbon fixation in photosynthetic organisms				
L1 to L2	*Cluster-17,798.0*	6.3318	0.0006952	Phosphoenolpyruvate carboxykinase (ATP)
L2 to L3	*Cluster-20,520.0*	−6.3406	0.03053	Ribulose bisphosphate carboxylase small chain
	*Cluster-23,656.0*	−6.1474	0.0089179	Ribulose-bisphosphate carboxylase small chain
L2 to L4	*Cluster-164.0*	−7.439	0.0009329	Ribulose bisphosphate carboxylase small chain
L3 to L4	*Cluster-23,656.0*	5.8291	0.034387	Ribulose-bisphosphate carboxylase small chain
	*Cluster-164.0*	−7.3099	0.0052386	Ribulose bisphosphate carboxylase small chain
Starch and sucrose metabolism				
L1 to L2	*Cluster-24,085.63483*	3.2081	0.003801	UDP-glycosyltransferase 76F1-like
L1 to L4	*Cluster-24,085.9268*	−2.4712	7.81E-05	Alpha,alpha-trehalose-phosphate synthase
	*Cluster-24,085.20752*	4.2334	1.38E-05	Glucose-1-phosphate adenylyltransferase
	*Cluster-24,085.45906*	2.3643	2.53E-05	1,4-alpha-glucan-branching enzyme
	*Cluster-24,085.42146*	2.0882	0.005112	Beta-amylase 3, chloroplastic-like
L2to L4	*Cluster-24,085.48054*	−1.4951	0.0051263	UDP-glucuronate 4-epimerase 6
	*Cluster-24,085.9268*	−1.877	0.0049111	Alpha,alpha-trehalose-phosphate synthase
	*Cluster-24,085.77617*	−6.2549	0.026686	UDP-glucuronate decarboxylase
	*Cluster-24,085.43359*	−2.5661	0.049914	Growth-regulating factor 1-like
	*Cluster-24,085.42146*	2.4966	0.0002086	Beta-amylase 3, chloroplastic-like

We searched our transcriptome for DEGs related to the carotenoid pathway and found one gene (*Cluster-9588.0*) that was upregulated under full sunlight conditions as compared to 50% as well as 12% light conditions. Additionally, we found two more unigenes (*Cluster-24,085.17524* and *Cluster-24,085.21006*) that were actually downregulated under full sunlight conditions as compared to 12% light. These genes are involved in the abscisic acid biosynthesis part of the carotenoid biosynthesis where the first gene controls the final step of xanthoxin formation while the second gene converts abscisate to dihydroxy-phaseic acid (Table 2; Additional Table 2; Table 4).

**Table 4:** DEGs enriched in plant hormone signal transduction pathway in *Asarum* leaves grown under different light intensities. L1, L2, L3 and L4 represent full sunlight, 50% sunlight, 28% sunlight and 12% sunlight, respectively

Treatment	Gene ID	Log2 fold change	Pvalue adjusted	Description
Plant hormone signal transduction				
L1 to L2	*Cluster-24,085.2657*	3.4089	0.035652	Ethylene-responsive TF 1B-like
L1 to L4	*Cluster-24,085.21051*	−1.6807	0.0015999	Auxin responsive SAUR protein
	*Cluster-24,085.68791*	−4.5239	3.15E-05	Xyloglucan:xyloglucosyl transferase TCH4
	*Cluster-24,085.68790*	−3.7161	0.0008913	Xyloglucan:xyloglucosyl transferase TCH4
	*Cluster-19,269.0*	−6.1483	0.011149	Auxin-responsive protein SAUR72
	*Cluster-24,085.34259*	1.9218	0.015288	Histidine-containing phosphotransfer protein 4
	*Cluster-24,085.18269*	−3.1107	0.015652	Xyloglucan:xyloglucosyl transferase TCH4
L2 to L4	*Cluster-24,085.21051*	−1.7653	1.48E-05	Auxin responsive SAUR protein
	*Cluster-24,085.68791*	−5.2357	4.67E-09	Xyloglucan:xyloglucosyl transferase TCH4
	*Cluster-24,085.68792*	−4.9808	3.62E-08	Xyloglucan:xyloglucosyl transferase TCH4
	*Cluster-24,085.68790*	−2.8511	0.014699	Xyloglucan:xyloglucosyl transferase TCH4
	*Cluster-19,269.0*	−4.8215	0.014835	Auxin-responsive protein SAUR72
	*Cluster-24,085.51860*	−1.4164	0.017439	Auxin-responsive protein IAA4
	*Cluster-24,085.34259*	2.3245	0.0024764	Histidine-containing phosphotransfer protein 4-like
	*Cluster-24,085.3665*	−2.2309	9.73E-09	Protein phosphatase 2C 37-like
	*Cluster-24,085.25146*	−1.5467	0.002965	TF MYC2
	*Cluster-24,085.41514*	−1.4164	0.017439	Auxin-responsive protein IAA4
	*Cluster-24,085.77156*	−1.886	0.032	Auxin early response protein SAUR41
	*Cluster-24,085.18269*	−4.2914	7.80E-28	Xyloglucan:xyloglucosyl transferase TCH4
	*Cluster-24,085.14689*	−3.7539	0.0021302	Auxin-responsive protein IAA25
	*Cluster-24,085.60271*	−1.8161	0.0065038	Jasmonate ZIM domain-containing protein
L3 to L4	*Cluster-24,085.68791*	−2.8209	0.022986	Xyloglucan:xyloglucosyl transferase TCH4
	*Cluster-24,085.34259*	2.624	0.011739	Histidine-containing phosphotransfer protein 4-like
	*Cluster-24,085.18269*	−2.3918	0.0027324	Xyloglucan endotransglucosylase/hydrolase protein 22
	*Cluster-24,085.2318*	−1.912	0.036532	Cyclin-D3–2

Our results demonstrated that ascorbate and aldarate metabolism was a significantly enriched pathway under the studied light conditions. Therefore, we searched for DEGs related to this pathway and found that two genes annotated as L-ascorbate oxidase were downregulated in plants grown under full sunlight conditions (Table 3). The first gene (*Cluster-24,085.74455*) was downregulated in *Asarum* leaves grown under full to 50%, full to 12, 50 to 12%, and 28 to 12% sunlight conditions. This gene controls the conversion of L-ascorbate to L-dehydro ascorbate. The second gene (*Cluster-24,085.74454*) was the only differentially expressed gene between full to 12% sunlight conditions and plays the same role as the first gene (Table 3; Additional Table 4). A UDP-glycosyltransferase 76F1-like gene (*Cluster-24,085.63483*) was upregulated in full sunlight grown *Asarum* leaves as compared to the ones grown under 50% light conditions. This gene converts UDP-D-glucuronate to D-glucuronate [39].

Carbon fixation is an important process in photosynthetic organisms, which affects carbon acquisition and biomass allocation. Light intensity has been reported to be an important factor in this regard [40]. We found that carbon fixation in photosynthetic organisms was one of the significantly enriched pathways under the studied light conditions (Additional Fig. 3). The upregulation of a phosphoenolpyruvate carboxykinase (ATP) gene (*Cluster-17,798.0*) in full sunlight grown leaves as compared to those grown in 50% sunlight conditions was consistent with the findings of a previous study in soybean [41]. Three genes annotated as ribulose-bisphosphate carboxylase small chain were also differentially regulated under low-light intensities i.e. *Cluster-20,520.0* in 50 to 28%, *Cluster-23,656.0* in 50 to 28% and 28 to 12%, and *Cluster-164.0* in 50 to 28% and 28 to 12% light conditions (Table 3). However, these three genes did not differentially express under full sunlight conditions.

Another key pathway i.e. starch and sucrose metabolism pathway has an important role in overall plant development and biomass accumulation [42]. Several genes involved in this pathway such as UDP-glycosyltransferase 76F1-like, Glucose-1-phosphate adenylyltransferase, 1,4-alpha-glucan-branching enzyme, and β-amylase 3, chloroplastic-like, were upregulated in higher light intensities. Some genes such as alpha,alpha-trehalose-phosphate synthase, UDP-glucuronate 4-epimerase 6, UDP-glucuronate decarboxylase, and growth-regulating factor 1-like were downregulated in high light intensity to low intensity. Only one of these four genes (alpha,alpha-trehalose-phosphate synthase) was differentially regulated in full sunlight conditions while the other three were differentially regulated between 50 to 12% light conditions (Table 3; Additional Table 5).

The processes of photosynthesis and biomass accumulation are affected by several pathways. Therefore, we searched for DEGs that have been reported in this regard. Stay-green genes regulate chlorophyll degradation during dark-induced senescence [43]. We noticed that one gene predicted as Stay-green protein (*Cluster-24,085.51159*) was upregulated in *Asarum* leaves grown under full sunlight grown leaves as compared to those grown in 50 and 12% light. Another predicted Stay-green gene (*Cluster-24,085.56669*) was upregulated between full to 12% sunlight conditions (Additional Table 2; Table 4). Among other DEGs, we observed differential regulation of ATP-dependent DNA helicase Q-like genes, cytochrome P450, oxaloacetate decarboxylase, ZINC INDUCED FACILITATOR-LIKE 1-like genes, subtilisin-like proteases, BURP domain protein RD22, protein trichome birefringence-like 38, and receptor-like protein kinases [44–46].

#### Differential regulation of genes related to hormones

We searched for DEGs related to hormone signaling pathways [47]. A gene (*Cluster-24,085.2657*) related to ethylene responsive factor in plant hormone signal transduction pathway was upregulated in full sunlight grown *Asarum* leaves as compared to 50% light treated leaves (Additional Table 2). A histidine-containing phosphotransfer protein (*Cluster-24,085.34259*) was also upregulated in these light conditions. This gene was also upregulated in treatment in low light treatment comparisons i.e. 50 to 12% and 28 to 12% light conditions (Table 4). On the other hand, we noticed the downregulation of two auxin responsive SAUR proteins (*Cluster-24,085.21051* and *Cluster-19,269.0*) and three xyloglucan:xyloglucosyl transferase TCH4s (*Cluster-24,085.68791*, *Cluster-24,085.68790*, and *Cluster-24,085.18269*) in full sunlight grown *Asarum* leaves as compared to those grown in 12% light conditions (Additional Table 4; Table 4). Both auxin responsive SAUR genes were also downregulated in *Asarum* leaves grown under 50% sunlight as compared to 12% sunlight conditions (Table 4; Additional Table 6). A similar pattern was observed for the expression of xyloglucan:xyloglucosyl transferase TCH4s. We noticed that a relatively higher number of genes were differentially expressed between 50 and 12% sunlight conditions as compared to full sunlight versus other three light conditions. Those genes were annotated as auxin-responsive protein IAA4, protein phosphatase 2C 37-like, TF MYC2, auxin-responsive protein IAA4, auxin early response protein SAUR41, auxin-responsive protein IAA25, and jasmonate ZIM domain-containing protein (Table 4; Additional Table 6).

#### Transcription factors active in regulation of gene expression under different light intensities in Asarum leaves

Our comparative transcriptome data showed the upregulation of only one TF i.e. AP2/ERF-ERF (*Cluster-24,085.2657*) in full sunlight grown *Asarum* leaves as compared to 50% light grown leaves. By comparing the full sunlight and 28% light conditions, three TFs i.e. two AP2/ERF-ERFs (*Cluster-24,085.12486* and *Cluster-24,085.17619*) and one WRKY (*Cluster-24,085.14452*) were downregulated while, one WRKY, one MYB, one, TAZ, and one OFP were upregulated. The AP2/EFF-ERF TF (*Cluster-24,085.12486*) was also downregulated in full to 28%, full to 12, 50 to 28%, and 50 to 12% sunlight conditions. Similarly, the WRKY TF (*Cluster-24,085.14452*) was also downregulated in 50 to 12% light conditions. Eight AP2/ERF-ERFs, one AP2/ERF-RAV, one C2H2, one MBF1, and one NAC were downregulated between 50 and 28% light conditions while only one bHLH was upregulated. A relatively larger number of TFs belonging to 24 families were differentially regulated. Finally, the transcriptome comparison between 28 and 12% light conditions showed the downregulation of TFs belonging to AP2/ERF-EFF, C2C2-DOF, HSF, ZF-HD, and MYB-related gene families (Additional Table 7). Interestingly, full sunlight comparisons with the three low light intensities resulted in the upregulation of some TFs while the comparisons between the low light conditions did not show upregulation of TFs except one bHLH (in 50 to 28% and 50 to 12%) (Table 5).

**Table 5:** List of differentially regulated transcription factors in *Asarum* leaves grown under different light intensities. L1, L2, L3 and L4 represent full sunlight, 50% sunlight, 28% sunlight and 12% sunlight, respectively

Treatment	Gene_id	Log2 Fold Change	Pvalue adjusted	TF family
L1 to L2	*Cluster-24,085.2657*	3.4089	0.035652	AP2/ERF-ERF
L1 to L3	*Cluster-24,085.12486*	−4.3794	3.59E-05	AP2/ERF-ERF
	*Cluster-24,085.14452*	−3.184	0.0020601	WRKY
	*Cluster-24,085.20439*	2.7652	0.0096977	WRKY
	*Cluster-24,085.17619*	−2.7775	0.018342	AP2/ERF-ERF
	*Cluster-13,720.1*	4.1745	0.020535	MYB
	*Cluster-24,085.66840*	3.2079	0.020558	TAZ
	*Cluster-24,085.18987*	3.438	0.028837	OFP
L1 to L4	*Cluster-24,085.12486*	−6.3006	6.07E-15	AP2/ERF-ERF
	*Cluster-24,085.10041*	−3.3336	6.31E-13	AP2/ERF-ERF
	*Cluster-24,085.27017*	−5.5571	6.31E-13	HSF
L2 to L3	*Cluster-24,085.12486*	−4.0405	1.08E-09	AP2/ERF-ERF
	*Cluster-24,085.1987*	−3.8493	8.26E-07	AP2/ERF-ERF
	*Cluster-24,085.17619*	−2.7933	5.62E-06	AP2/ERF-ERF
	*Cluster-24,085.21290*	−2.1018	2.75E-05	AP2/ERF-ERF
	*Cluster-24,085.2466*	−2.34	3.19E-05	MADS-MIKC
	*Cluster-24,085.20260*	−2.7511	4.87E-05	C2H2
	*Cluster-24,085.20026*	−2.5195	6.62E-05	AP2/ERF-ERF
	*Cluster-24,085.10041*	−2.0953	0.00012223	AP2/ERF-ERF
	*Cluster-24,085.42603*	−2.0689	0.00017711	AP2/ERF-ERF
	*Cluster-24,085.37030*	−1.6079	0.00026526	MBF1
	*Cluster-24,085.23378*	−1.7439	0.0004069	AP2/ERF-ERF
	*Cluster-24,085.23170*	−1.6109	0.0019938	NAC
	*Cluster-24,085.20351*	−1.6613	0.0060609	AP2/ERF-ERF
	*Cluster-24,085.19577*	−1.0273	0.012246	AP2/ERF-RAV
	*Cluster-24,085.52171*	2.1066	0.041305	bHLH
L2 to L4	*Cluster-19,135.0*	−5.9595	8.57E-05	AP2/ERF-ERF
	*Cluster-24,085.10041*	−3.8815	3.67E-37	AP2/ERF-ERF
	*Cluster-24,085.10373*	−2.9536	5.46E-08	AP2/ERF-ERF
	*Cluster-24,085.12486*	−5.961	1.15E-30	WRKY
	*Cluster-24,085.1287*	−9.4367	0.00089839	AP2/ERF-ERF
	*Cluster-24,085.14452*	−3.4296	0.00013736	WRKY
	*Cluster-24,085.14689*	−3.7539	0.0021302	AUX/IAA
	*Cluster-24,085.14837*	−1.0913	0.025532	AP2/ERF-ERF
	*Cluster-24,085.15903*	−2.7791	0.0008352	MYB
	*Cluster-24,085.16202*	−2.3493	1.96E-05	MYB
	*Cluster-24,085.1707*	−3.3558	6.79E-05	AP2/ERF-ERF
	*Cluster-24,085.17343*	−2.382	8.54E-07	AP2/ERF-ERF
	*Cluster-24,085.17619*	−3.8718	3.91E-20	AP2/ERF-ERF
	*Cluster-24,085.18098*	−2.036	0.011069	C2H2
	*Cluster-24,085.18531*	−2.2255	0.0014194	MYB-related
	*Cluster-24,085.19218*	−2.5795	0.00041875	NAC
	*Cluster-24,085.19220*	−2.3685	3.27E-10	AP2/ERF-ERF
	*Cluster-24,085.19364*	−3.1888	0.039496	GARP-G2-like
	*Cluster-24,085.19396*	−1.7638	3.92E-10	C2C2-GATA
	*Cluster-24,085.19455*	−3.2023	7.25E-08	WRKY
	*Cluster-24,085.19577*	−1.6049	3.33E-05	AP2/ERF-RAV
	*Cluster-24,085.19626*	−2.1474	0.02618	AP2/ERF-ERF
	*Cluster-24,085.1987*	−5.5105	5.08E-16	AP2/ERF-ERF
	*Cluster-24,085.20026*	−4.059	9.32E-25	AP2/ERF-ERF
	*Cluster-24,085.20260*	−4.4895	7.77E-25	C2H2
	*Cluster-24,085.20351*	−2.9081	1.19E-16	AP2/ERF-ERF
	*Cluster-24,085.20438*	−1.8845	0.0029452	MYB
	*Cluster-24,085.21290*	−2.9988	6.58E-25	AP2/ERF-ERF
	*Cluster-24,085.21518*	−3.4887	0.001799	AP2/ERF-ERF
	*Cluster-24,085.21683*	−1.3931	0.0078483	Others
	*Cluster-24,085.21894*	−2.4352	2.32E-06	WRKY
	*Cluster-24,085.21936*	−2.0129	0.0056915	WRKY
	*Cluster-24,085.23378*	−3.1779	3.20E-31	AP2/ERF-ERF
	*Cluster-24,085.23829*	−1.1127	0.0333	bHLH
	*Cluster-24,085.2477*	−1.6509	0.018848	C2C2-Dof
	*Cluster-24,085.25146*	−1.5467	0.002965	bHLH
	*Cluster-24,085.2521*	−3.6674	1.43E-10	WRKY
	*Cluster-24,085.27017*	−5.3399	1.20E-11	HSF
	*Cluster-24,085.27420*	−2.0865	0.0003884	LOB
	*Cluster-24,085.32540*	−1.9852	0.0004087	C2H2
	*Cluster-24,085.3305*	−1.9823	1.62E-12	HB-HD-ZIP
	*Cluster-24,085.35957*	−2.2082	7.30E-11	WRKY
	*Cluster-24,085.36255*	−1.0905	0.044329	HB-HD-ZIP
	*Cluster-24,085.37030*	−2.9731	5.77E-06	MBF1
	*Cluster-24,085.38470*	−1.3752	0.013487	GRAS
	*Cluster-24,085.41221*	−1.4224	0.0035887	C3H
	*Cluster-24,085.4136*	−1.7074	0.0025093	C2C2-GATA
	*Cluster-24,085.41514*	−1.4164	0.017439	AUX/IAA
	*Cluster-24,085.42603*	−3.2229	6.46E-18	AP2/ERF-ERF
	*Cluster-24,085.44733*	−3.4652	6.20E-11	AP2/ERF-ERF
	*Cluster-24,085.45927*	−1.6676	0.038415	HSF
	*Cluster-24,085.4609*	−1.6993	0.018848	GNAT
	*Cluster-24,085.51860*	−1.4164	0.017439	AUX/IAA
	*Cluster-24,085.52171*	2.0002	0.035938	bHLH
	*Cluster-24,085.546*	−3.6975	3.41E-15	zf-HD
	*Cluster-24,085.55958*	−1.5396	0.0008922	C2C2-GATA
	*Cluster-24,085.60271*	−1.8161	0.0065038	Tify
	*Cluster-24,085.60939*	−2.6318	0.00011965	NAC
	*Cluster-24,085.61360*	−2.8417	5.75E-13	HSF
	*Cluster-24,085.64005*	−1.1783	0.028667	LIM
	*Cluster-24,085.72259*	−2.2086	0.0072876	B3-ARF
	*Cluster-24,085.77102*	−1.3448	0.032949	TCP
	*Cluster-24,085.77333*	−4.1789	0.007682	AP2/ERF-ERF
	*Cluster-24,085.77948*	−6.5356	4.01E-11	AP2/ERF-ERF
	*Cluster-24,085.809*	−6.9567	1.55E-10	AP2/ERF-ERF
	*Cluster-24,085.995*	−5.6805	0.0042634	OFP
	*Cluster-24,730.0*	−4.3569	0.022909	AP2/ERF-ERF
	*Cluster-4248.0*	−5.3527	0.039977	MYB-related
L3 to L4	*Cluster-24,085.10041*	−1.7807	0.00096256	AP2/ERF-ERF
	*Cluster-24,085.10095*	−5.628	0.0075127	C2C2-Dof
	*Cluster-24,085.17343*	−1.7345	0.0064897	AP2/ERF-ERF
	*Cluster-24,085.19626*	−2.0562	0.045191	AP2/ERF-ERF
	*Cluster-24,085.27017*	−3.4274	0.00081077	HSF
	*Cluster-24,085.44733*	−2.0545	0.0023397	AP2/ERF-ERF
	*Cluster-24,085.546*	−2.2331	0.024219	zf-HD
	*Cluster-24,085.809*	−3.2375	0.031137	AP2/ERF-ERF
	*Cluster-4248.0*	−7.0589	0.014273	MYB-related

### Quantitative real-time PCR (qRT-PCR) analysis

**Fig. 4:**
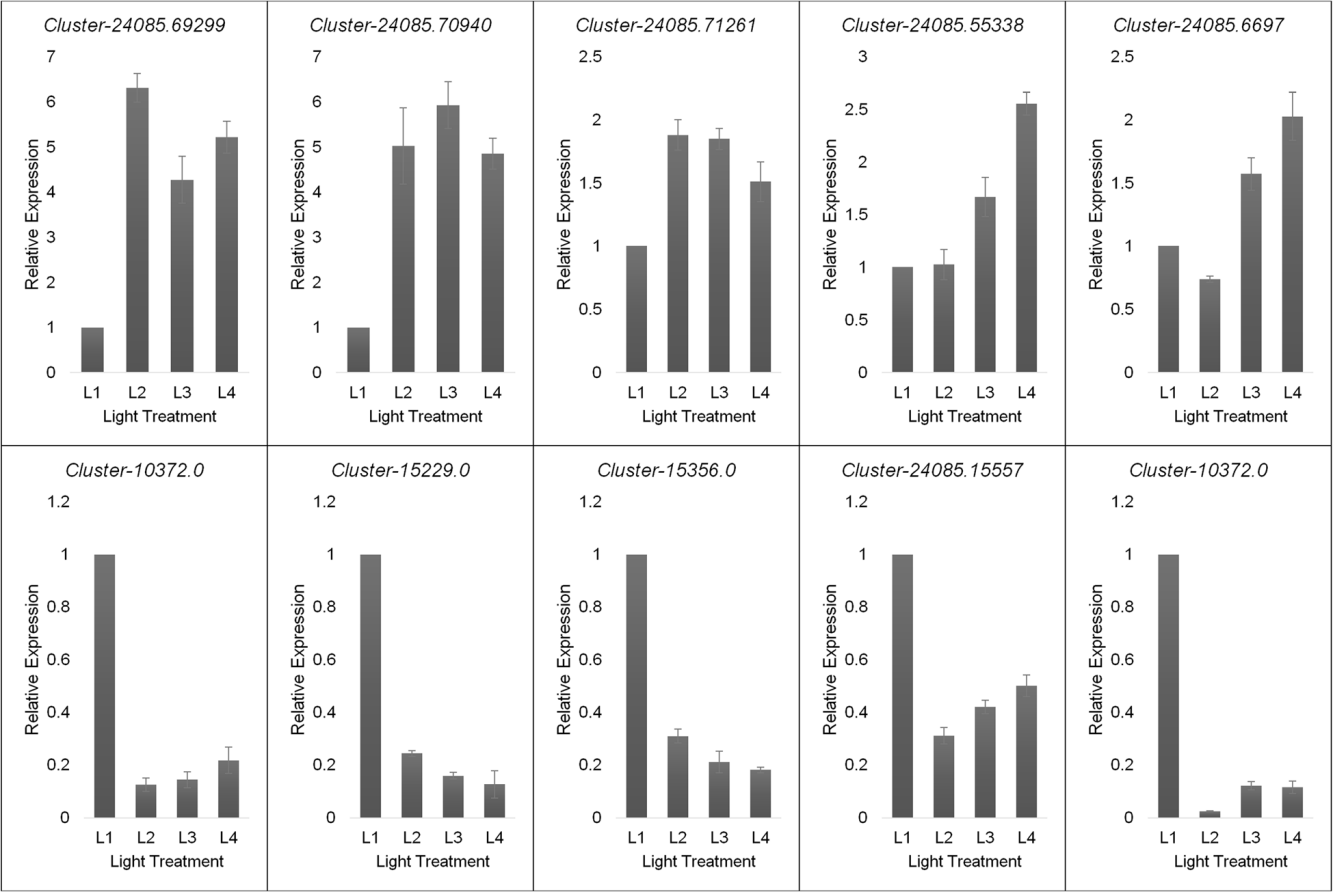
qRT-PCR validation of the selected differentially expressed genes (DEGs) in Asarum leaves. L1, L2, L3 and L4 represent full sunlight, 50% sunlight, 28% sunlight and 12% sunlight, respectively

We validated the expression profiles of ten *Asarum* genes (Fig. 4). The selection of the genes was random to achieve the objective of this analysis i.e. validation of the expression results observed in transcriptome analysis. The *Actin* gene was used as the internal control to standardize the data. Among the tested genes, five were upregulated and five were downregulated in full sunlight conditions as compared to low light conditions. The results of the qRT-PCR showed similar expression patterns as recorded in the transcriptome analysis confirming the reliability of our RNA-Seq data.

## Discussion

### Effect of light on volatile oil biosynthesis in Asarum leaves

**Fig. 5:**
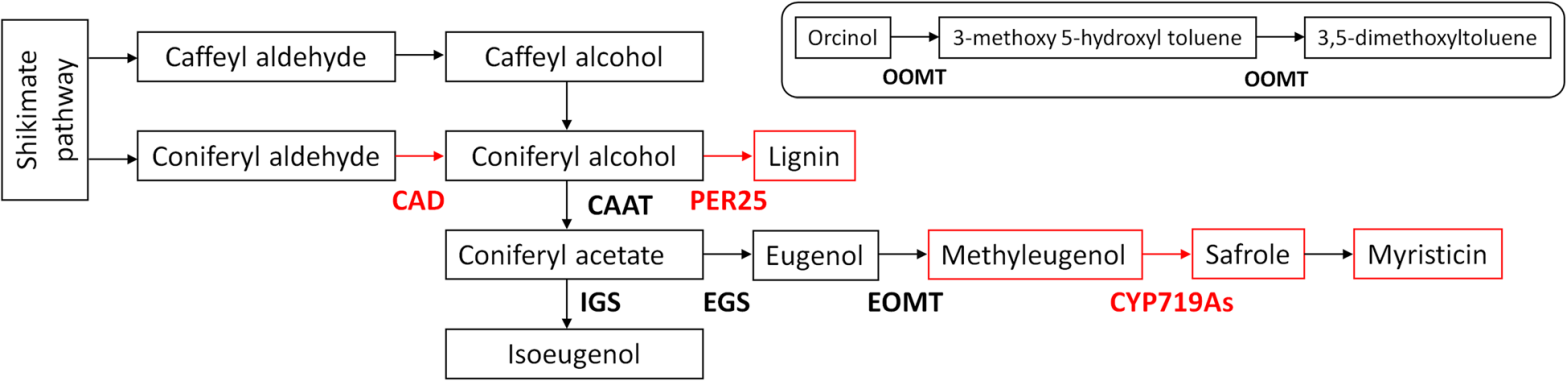
Important steps of the phenylpropanoid pathway in the formation of methyleugenol, safrole, and myristicin and the formation of toluene. Where CAD = cinnamyl alcohol dehydrogenase, CAAT = coniferyl alcohol acyl transferase, PER25 = peroxidase 25, IGS = isoeugenol synthase, EGS = eugenol synthase, EOMT = eugenol O-methyltransferase, and CYP719As = cytochrome-450719As. Red colour shows the upregulation of DEGs in *Asarum* leaves in full sunlight conditions

*Asarum* is a sciophyte that grows in habitats with reduced sunlight. Volatile oil is an essential component of its extracts that have importance as antifungal, antibacterial, antidepressant, and anxiolytic effects. Despite its medicinal importance and availability, only a limited knowledge exists about the effect of light conditions on the biosynthesis of volatile oil [4]. Hence, it is important to understand if the *Asarum* volatile oil components’ are affected by growing this plant in full sunlight conditions. Recent work has shown that the composition of volatile oil detected through LC-MS was different under different light conditions but the total volatile oil content did not significantly differ among the *Asarum* plants [7]. Our results demonstrated that the individual percent content of each of the four components showed the positive effect of full sunlight conditions as compared to reduced light intensities. To understand the effect of light on the phenylpropanoid pathway, we searched for DEGs that were significantly enriched under the tested conditions. The established phenylpropanoid pathway has shown that coniferyl aldehyde is converted into coniferyl alcohol by the reaction of CAD which is an important step for the subsequent formation of methyleugenol, safrole, and myristicin [25] (Fig. 5). Two CADs were highly expressed in full sunlight conditions; one in full to 50% and one in full to 28% sunlight conditions. Therefore, the higher accumulation of these three components in the full sunlight conditions suggests that higher light intensities affect the volatile oil formation by increasing the CAD expression. Alternatively, the coniferyl alcohol can also be converted to lignin by the action of peroxidases. Therefore, the upregulation of CADs in *Asarum* leaves grown under higher light intensities (full sunlight) would lead to higher lignin content. In this regard, the upregulation of a peroxidase responsible for the lignin formation suggested that under higher light intensities, higher lignin content is produced in the *Asarum* leaves. This observation is similar with those of heliophytes where it is known that leaf lignin content increases with the increasing light intensities [48]. Once, the methyleugenol is formed, it can further be converted into safrole and then into myristicin. The formation of safrole is under the control of cytochrome p450s (specifically CYP719As) [25]. We found the upregulation of two CYP719As in higher light intensity (50%) to lower light intensity (12%) (Additional Table 5) which may explain the higher safrole content and subsequently myristicin content in high light intensity. The fourth major component i.e. toluene is synthesized from orcinol by the action of orcinol-OMTs in rose. However, very little is known about the biosynthesis of orcinol, which is hydroxylated in position 3 and 5 [27]. Some studies have reported that orcinol is biosynthesized by the monolignol biosynthetic pathway [49]. A recent study on *Rhododendron dauricum* L. demonstrated that orcinol is produced from tetraketide non-enzymatically by decarboxylative aldol condensation [50]. In our results, no OMT was differentially regulated between full sunlight and 50% or 28% light conditions. However, two OMTs (one protein L-isoaspartyl methyltransferase (PIMT) and one trans-resveratrol di-O-methyltransferase (ROMT)) were differentially regulated between full and 12% sunlight conditions (Additional Table 4). PIMT was downregulated while the ROMT was upregulated. ROMT is implicated in stilbene pathway where it catalyses the biosynthesis of pterostilbene from resveratrol in grapevine and our KEGG pathway mapping was also in agreement with this study [51]. Based on the percent content observations, it is found that somehow light affects toluene content but the pathway and the genes involved in this increase needs specific studies or a different approach. Altogether, these results propose that the light treatment had a positive effect on the contents of the four volatile oil component.

### Effect of full sunlight conditions on photosynthesis and biomass accumulation

Light intensity and quality are the most critical environmental factors for crop physiology and biochemistry. *Asarum* being a sciophyte grows in shady environment and there have been some studies which tried to address the effect of full sunlight on photosynthesis and related parameters. Our study also discusses the transcriptional changes within *Asarum* leaves related to photosynthesis and biomass accumulation. It is known that higher plants’ chloroplasts convert light into biological energy by employing electron transport chain and Calvin-Benson cycle. This process uses genes and proteins of both nuclear and chloroplast origins and their expression is highly dynamic and is primarily influenced by light [29]. When *Asarum* leaves were grown under full sunlight conditions, the photosynthesis-antenna proteins were downregulated as compared to the studied low light treatments i.e. 50, 28, and 12% light conditions (Additional Tables 2, 3, and 4; Table 2). Previous research on photosynthesis has shown that photosynthetic electron transport rates are light saturated at approximately 1/4th of full sunlight intensity. This is due to large optical cross section of light harvesting antenna complexes that capture photons relatively faster (~ 10-fold faster) than the rate-limiting step in electron transport. Therefore, this downregulation of several antenna proteins in full sunlight conditions as compared to low light intensities is possibly due to this phenomenon.

Carotenoids play essential role in photosynthesis and photoprotection and studies have demonstrated that carotenoid biosynthesis is regulated by light [52, 53]. Transcriptome comparison showed the upregulation of a β-carotene 3-hydroxylase in full sunlight conditions which converts α-carotene to zeinoxanthin, which is finally converted into O-lutein. It also directly converts α-carotene to O-lutein with the help of another enzyme LUT1 [54]. It is the most abundant carotenoid and the only ligand for site L1 in light-harvesting complex proteins. The upregulation of this gene might be the increasing demand to quench the antenna complex in *Asarum* leaves under full sunlight conditions similar to previous report in Arabidopsis [55]. In addition to this, the downregulation of one gene controlling the final step in the xanthoxin formation in full sunlight versus 12% sunlight conditions might also be the part of *Asarum* leaves’ photoprotective strategy under full sunlight conditions (Table 2; Additional Table 2; Table 4). So, the transcriptomic observations under full sunlight conditions related to antenna proteins as well as carotenoids suggest that *Asarum* leaves develop a photoprotective strategy under excess light conditions.

Because a moderate reduction in antenna size results in increased photosynthesis and resultantly higher biomass accumulation, so the observation of different genes related to biomass accumulation under full sunlight conditions in *Asarum* was expected. In this regard, the downregulation of L-ascrobate oxidases in full sunlight conditions could be important because it is previously known that high light intensity induces higher ascorbate levels in the leaves. It could be possibly due to low ascorbate mutation and needs further specific investigation. Nevertheless, It is documented that ascorbate mediates regulation of plant growth and plays important role in biomass accumulation in plants [56]. The upregulation of phosphoenylpryrovate carboxykinase (ATP) in full sunlight is quite interesting because it is reported that this enzyme is less active during the day time largely by dephosphorylation of the enzyme, which makes it less sensitive to stimulatory metabolites and more sensitive to inhibitory metabolites, such as malate. So further studies on this gene and its regulation in day time will be needed to understand its function in *Asarum* leaves [57]. Apart from these adaptations, plants also use various strategies to assimilate carbohydrates in vegetative organs. In this regard, one important strategy is to stay-green. This was recently discovered in maize where maize plants stay green for a longer time [36]. Furthermore, it is known that stay-green proteins regulate chlorophyll degradation in dark-induced senescence [43]. The upregulation of two stay-green genes in full sunlight conditions suggests that *Asarum* leaves, when provided with full sunlight, tend to accumulate higher biomass.

### Effect of full sunlight conditions on hormone-signal transduction

Light is vital for plant growth and development; it provides energy for photosynthesis, but also affects overall plant growth and development through the regulation of endogenous plant hormones [58]. Studies in Arabidopsis have reported that a retrograde signaling is associated with the redox and hormonal pathways. The study demonstrated that AP2/ERFs show a quick upregulation in plants moved from dark to light. However, this response is for a very short time and in full sunlight conditions, the expression is reduced subsequently. Therefore, when the immediate response is finished for the transition from dark to light, under regular sunlight, the expression of these TFs should decrease. The downregulation of AP2/ERFs in higher light intensities as compared to the lower light intensities suggests that a similar response exists in *Asarum* leaves (Table 5) [59]. However, we noticed that one AP2/ERF (*Cluster-24,085.2657*) was upregulated in full compared to 50% sunlight conditions. This was quite different from overall TFs of the same family and needs further specific investigation. The histidine-containing phosphotransfer protein which was upregulated under higher light conditions as compared to lower light conditions is implicated in cytokinin pathway where it plays an important role in cell division and shoot initiation [60]. Thus, this gene might be increasing the biomass through increasing cell division and should be studied further. Concerning this observation, the downregulation of xyloglucan:xyloglucosyl transferase TCH4s is interesting because cell wall modifications are needed for cell division [61]. Therefore, enzymes involved in xyloglucan modification may affect cell wall characteristics and, ultimately, cell shape and plant form [62, 63]. Under low light conditions, plants experience increased auxin biosynthesis and transport as well as auxin sensitivity are enhanced [64]. That means, when sufficient light is available for normal growth of the plant, it should do the reverse. In our transcriptome data of low light and full sunlight conditions, the downregulation of auxin responsive SAUR genes suggests that *Asarum* leaves, when grow under low light conditions, increase auxin sensitivity by upregulating these genes and when the light is sufficient (full sunlight conditions), auxin sensitivity is no more needed [65]. Following the above statement that the auxin sensitivity is not needed under full sunlight conditions, the downregulation of AUX/IAAs (Auxin/indole-3-acetic acid) further confirms this adaptation in *Asarum* leaves [64].

## Conclusion

In this study, high-throughput sequencing of *Asarum* transcriptome was performed under different light conditions. In-depth transcriptome analysis allowed us to identify the expression levels of key genes involved in the volatile oil biosynthesis. Specifically, we targeted genes involved in the biosynthesis of four major volatile oil components i.e. methyleugeol, safrol, myristicin, and toluene biosynthesis. The variation of the major genes in addition to the biochemical data of the four components demonstrated that high light conditions enrich *Asarum* volatile oil with these four components. We further studied the expression of genes related to photosynthesis under full sunlight conditions, which revealed complexity of gene expression in the studied sciophyte. We also found the expression of hormone-signaling pathway related genes in the light of previous studies. Our transcriptome data represent the first genomic resource of *Asarum* under different light conditions and lays the foundation for further research aiming at enriching important bioactive metabolites in medicinal plants using genetics, genomics, and biotechnology methods. We conclude that full sunlight conditions help *Asarum* to increase volatile oil biosynthesis. The light intensity under full sunlight conditions is managed by the regulation of antenna proteins and *Asarum* leaves accumulate biomass when grown in full sunlight. Based on these conclusions, it could be recommended that *A. heterotropoides* can be grown in full sunlight conditions for increased biomass and volatile oil biosynthesis.

## Methods

### Plant material

**Fig. 6:**
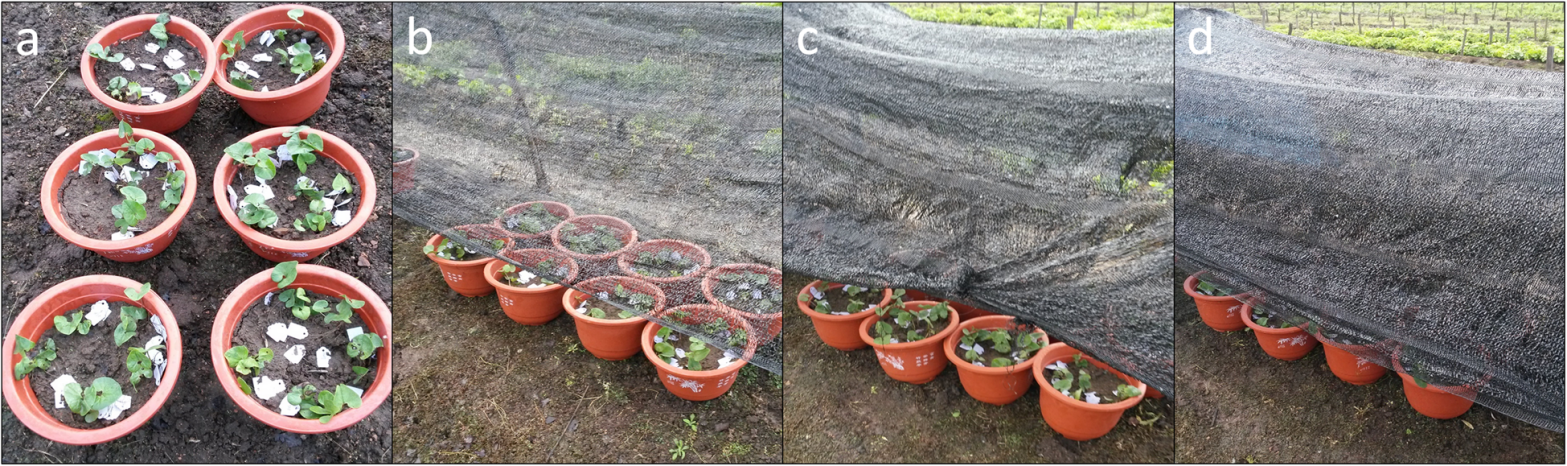
Light treatments **a)** full sunlight (L1), **b)** 50% sunlight (L2), **c)** 28% sunlight (L3), **d)** and 12% sunlight (L4)

*Asarum* [*A. heterotropoides* Fr. Schmidt var. *mandshuricum* (Maxim.) Kitag.] cultivar zhongnong xixin-1 was used as plant material in this study. For plant material, no voucher has been deposited in a genebank. The formal identification of the plant materials was undertaken by the corresponding author of this article (Zhiqing Wang). The plants were originally collected from the wild in June 2018 and kept at the Medicinal Herbs Garden of Jilin Agricultural University. No permissions are necessary to collect such samples. In October 2019, we transplanted ~ 250 four-years old dormant seedlings into 30 cm diameter and 40 cm tall pots filled with humus (pH = 6.28). All pots were then placed at Medicinal Herbs Garden of Jilin Agricultural University (43.80′N, 125.42′E) under normal growing condition. In the next year, soon after the leaves appeared (second week of May 2019), we subjected the plants to different light treatments for 21 days; it is the time when *A. heterotropoides* leaves expand and it is known that the diurnal net photosynthetic rate increases when grown under variable light conditions [38]. The daytime temperature during 21 days treatment was 27.4–31 °C. Treatments consisted of full sunlight (L1), 50% sunlight; one layer of black nylon net providing 50% shade (L2), 28% sunlight; two layers of nylon net each providing ~ 50% shade (L3), and 12% sunlight; three layers of black nylon net each providing ~ 12% shade (L4), and were conducted in net houses (Fig. 6). Use of nets for the shade treatment was followed as reported and established in other studies [66, 67]. Average day length for the experimental period was 15 ± 1.5 h. Each treatment involved 24 pots with three replications and each pot contained 4–5 plants. For each replication, 6–8 leaves were randomly collected for volatile oil constituent analyses. For RNA extraction the 3–4 randomly selected leaves were harvested from each treatment, washed and stored immediately at − 80 °C. We used only leaves which were free from worms or any pathogen.

### Volatile oil constituent analysis by gas chromatography-mass spectrometry

After treatment, the leaves were harvested, cleaned, and placed in the shade with good ventilation. The dried leaves were ground into powder and subjected to hydrodistillation for 6 h. 10 μL of volatile oil was drawn from the solution and diluted 50 times using petroleum ether. 1 μL of this diluted sample was analyzed by gas chromatography-mass spectrometry. A HP-5MS highly polar capillary column (30 m × 0.32 mm × 0.25 μm, Hewlett-Packart, Palo Alto, CA, USA) coated with a 100% polyethylene glycol stationary phase was used. The following oven temperatures and times were used: 40 °C (held for 2 min), raised from 40 °C to 160 °C at a rate of 2.5 °C/min, from 160 °C to 280 °C at a rate of 8 °C/min, finally, held at 280 °C for 10 min. The injection temperature was set at 280 °C. Helium was used as the carrier gas with a flow rate of 1.0 mL/min. An injection volume of 1 μL was used with a split ratio of 100:1. The mass spectrometer was operated under a mode of electron impact (EI) at 70 eV with the scan ranges were between 30 and 550 amu. The ion source temperature was maintained at 230 °C and the quadrupole at 150 °C. Compound identification was done by comparing the NIST library data of the peaks with those reported in literature, and mass spectra was compared with peaks from literature data. Percentage composition was computed from GC peak areas on with DB-5 ms column without applying correction factors.

### RNA isolation and library preparation

Total RNA from the 6–8 leaves was extracted using Qiagen RNeasy mini kit (Qiagen, USA) following the manufacturer’s protocol and was pooled and considered as one replicate. For each treatment, three replicates were processed separately. The concentration of the RNA was measured by using a NanoPhotometer spectrophotometer (IMPLEN, CA, USA) and the purity was assessed by using RNA Nano 6000 Assay Kit of the Agilent Bioanalyzer 2100 system (Agilent Technologies, CA, USA). Sequencing libraries were created using NEB Next Ultra RNA Library Prep Kit (NEB, USA) following manufacturer’s instructions. Briefly, the mRNA was purified from 1.5 μg total RNA of each of three replicate using poly-T oligo-attached magnetic beads. Subsequently, the fragmentation buffer was used to break the RNA into short fragments, and the short-fragment RNA was used as a template to synthesize the first strand cDNA with random hexamer primer and M-MuLV Reverse Transcriptase (RNase H^−^), followed by buffer, dNTPs (dUTP, dATP, dGTP, and dCTP). Second strand cDNA synthesis was subsequently performed using DNA Polymerase I and RNase H and the double-stranded cDNA was purified using AMPure XP beads (Beckman Coulter, Beverly, USA). The purified double-stranded cDNA was subjected to terminal repair, A tail was added, and the sequencing linker was ligated, and then AMPure XP beads were used for fragment size selection, and finally PCR enrichment was performed to obtain a final cDNA library. Library quality was initially quantified using Qubit 2.0 using the 2100 to test the insert size of the library followed by accurately quantifying the effective concentration of the library (> 2 nM) by q-PCR. Finally, twelve paired-end cDNA libraries with an insert size of 300 bp were constructed for transcriptome sequencing and sequenced on Illumina HiSeq platform (Illumina Inc., San Diego, CA, USA) by Novogene, Beijing, China (https://en.novogene.com/).

### De novo assembly, functional annotation, classification and metabolic pathway analysis

The raw reads were filtered to obtain clean reads. Adapter sequences were trimmed, low quality reads containing > 50% bases with a Phred quality score ≤ 20 were removed, and reads with unknown nucleotides (more than 10% ambiguous residues N) using the FastQC tool (http://www.bioinformatics.babraham.ac.uk/projects/fastqc/) [68]. To stitch clean reads, Trinity was used (Version r20140717, [69]) and Trinity spliced transcript sequences were used as reference sequences for subsequent analyses. For hierarchical clustering, Corset was used (https://code.google.com/p/corset-project/). The assembled unigenes were then aligned with various databases such as KEGG [70], GO [71], Clusters of Orthologous Groups (COG) [72], PfAM (protein family), Swissprot [73], NR (NCBI non-redundant protein sequences) [74], KOG [75], and NT (NCBI non-redundant nucleotide sequences). The software KAAS (Moriya et al., 2007) was employed to get the unigene KEGG orthology with a threshold of E-value = 1.0 × 10^− 10^. For Swiss-Prot (E-value = 1.0 × 10^− 5^), KOG/COG (E-value = 1.0 × 10^− 3^), and NR (E-value = 1.0 × 10^− 5^) annotation we employed diamond V 0.8.22, respectively. For Nt annotation, NCBI BLAST 2.2.28+ was used with a threshold of E-value = 1.0 × 10^− 5^. The analogs of the unigene amino acid sequences were searched against the Pfam database [76] using HMMER tool [77] with a threshold of E-value = 0.01. For GO annotation was performed using Blast2GO V2.5 with a threshold E-value = 1.0 × 10^− 6^. The sequenced reads were compared with the unigene library using Bowtie2 with default parameters [78], and the level of expression was estimated in combination with RSEM [79]. The gene expression level was determined according to the Fragments Per Kilobase of transcript sequence per Millions base pairs sequenced (FPKM). For overall RNA-seq quality assessment, Pearson correlation check between samples (and treatments) was performed.

### Differential expression and enrichment analysis

The read count was normalized and DESeq R package (1.10.1) [80] was used to determine the differential expression genes (DEGs) between the treatments (I-IV) using a model based on the negative binomial distribution. The resulting *P* values were adjusted using the Benjamini and Hochberg’s approach for controlling the false discovery rate [81] and FDR correction set at *p* < 0.05. GO enrichment analysis was performed using the topGO method based on the wallenius noncentral hypergeometric distribution with *p* < 0.05 [82]. KEGG pathway enrichment analysis of the DEGs was done using KOBAS2.0 [83]. The FDR correction was employed (*P* < 0.05) to reduce false positive prediction of enriched GO terms and KEGG pathways.

### Identification of transcription factors

To perform the identification of transcription factors (TFs) in *A. heterotropoides* transcriptome data, we used iTAK software. The basic principal is to use the TF family and rules defined in the database to identify TF by hmmscan. TF identification and classification methods were followed as described earlier [84, 85].

### Quantitative RT-PCR analysis

Ten DEGs, characterized by interesting expression profiles in different shade treatments were selected for qRT-PCR. First strand cDNAs was synthesized from 100 ng of total RNA using the High Capacity cDNA Reverse Transcription Kit (Applied Biosystem). Primers were designed using Primer3 Software (http://frodo.wi.mit.edu/primer3/; Additional Table 8) and the specificity was checked by blasting their sequences in the NCBI database. The *Actin* constitutively expressed gene was used as reference gene [5]. All qRT-PCR reactions were carried out on a Rotor-Gene 6000 machine (Qiagen) with the following thermal cycling profile: 50 °C for 2 min and 95 °C for 2 min, followed by 40 cycles at 95 °C for 3 s and 60 °C for 30 s. Melting curve analysis was performed to verify single product amplification with temperature ranging from 55 to 95 °C by increasing of 1 °C every step. All reactions were performed in a total volume of 10 μl containing 30 ng of cDNA, 5 μl 1 × SYBR® Select Master Mix (Applied Biosystem) and 0.2 μl (20 μM) of each primer. For each sample, two biological replicates were analyzed in independent runs and a no-template control was included for each gene. Intra-assay variation was evaluated by performing all reactions in triplicate. The quantification cycle (Cq) was automatically determined using Rotor-Gene 6000 Series Software, version 1.7 as reported earlier [86].

## Supplementary Information

**Additional file 1: Table 1**. Summary of de novo transcriptome sequencing output. L1, L2, L3 and L4 represent full sunlight, 50% sunlight, 28% sunlight and 12% sunlight, respectively. **Table 2**. Differentially expressed genes in *Asarum* leaves grown under full and 50% sunlight conditions. **Table 3**. Differentially expressed genes in *Asarum* leaves grown under full and 28% sunlight conditions. **Table 4**. Differentially expressed genes in *Asarum* leaves grown under full and 12% sunlight conditions. **Table 5**. Differentially expressed genes in *Asarum* leaves grown under 50 and 28% sunlight conditions. **Table 6**. Differentially expressed genes in *Asarum* leaves grown under 50 and 12% sunlight conditions. **Table 7**. Differentially expressed genes in *Asarum* leaves grown under 28 and 12% sunlight conditions. **Table 8**. List of primers used for qRT-PCR analysis.

**Additional file 2: Figure 1**. Overview of transcriptome assembly data showing the size distribution of transcripts. **Figure 2**. Volcano plots of DEGs between treatments a) I vs II b) I vs III, c) I vs IV, d) II vs III, e) II vs IV, and f) III vs IV. I, II, III and IV represent full sunlight, 50% sunlight, 28% sunlight and 12% sunlight, respectively. **Figure 3**. KEGG enrichment analysis of DEGs between treatments a) I vs II b) I vs III, c) I vs IV, d) II vs III, e) II vs IV, and f) III vs IV. I, II, III and IV represent full sunlight, 50% sunlight, 28% sunlight and 12% sunlight, respectively.

## Data Availability

The RNA-seq data has been submitted to NCBI SRA: PRJNA603253.

## Abbreviations

ATP: Adenosine triphosphate
bHLH: Basic helix-loop-helix
C2H2: Cys_2_His_2_ zinc finger motifs
CAAT: Coniferyl alcohol acyl transferase
CAD: Cinnamyl alcohol dehydrogenase
cDNA: Complementary DNA
CYP719As: Cytochrome-450719As
DEG: Differentially expressed gene
EGS: Eugenol synthase
EOMT: Eugenol O-methyltransferase
ERF: Ethylene response factor
FPKM: Fragments per kilobase of transcript per million fragments mapped
Gb: Giga bite
GC-MS: Gas chromatography-mass spectrometery
GO: Gene ontology
HSF: Heat shock factors
IAA: Indole acetic acid
IGS: Isoeugenol synthase
KEGG: Kyoto encyclopedia of genes and genomes
KOG: Ortholog groups
L1: Full sunlight
L2: 50% sunlight
L3: 28% sunlight
L4: 12% sunlight
LC-MS: Liquid chromatography-mass spectrometry
LUT1: Lutein 1
MBF: Mlu1-binding factor
NAC: NAM, ATAF, AND CUC transcription factor
NR: Non-redundant
NT: Nucleotide
OMT: O-methyltransferases
PCR: Polymerase chain reaction
PER25: Peroxidase 25
PIMT: Protein L-isoaspartyl methyltransferase
ROMT: Trans-resveratrol di-O-methyltransferase
SAUR: Small auxin up-regulated RNA
SD: Standard deviation
TCH4: Touch 4, xyloglucan endotransglycosylase
TF: Transcription factor
ZF-HD: Zinc-finger-homeodomain
