# Supplementary material for: Transcriptional response of Asarum heterotropoides Fr. Schmidt var. mandshuricum (Maxim.) Kitag. leaves grown under full and partial daylight conditions
Source: BMC Genomics. 2021 Jan 6;22:16. doi: 10.1186/s12864-020-07266-7 (PMC7788892; doi:10.1186/s12864-020-07266-7)
Supplement: Supplementary file 2 — Additional file 2: Figure 1. Overview of transcriptome assembly data showing the size distribution of transcripts. Figure 2. Volcano plots of DEGs between treatments a) I vs II b) I vs III, c) I vs IV, d) II vs III, e) II vs IV, and f) III vs IV. I, II, III and IV represent full sunlight, 50% sunlight, 28% sunlight and 12% sunlight, respectively. Figure 3. KEGG enrichment analysis of DEGs between treatments a) I vs II b) I vs III, c) I vs IV, d) II vs III, e) II vs IV, and f) III vs IV. I, II, III and IV represent full sunlight, 50% sunlight, 28% sunlight and 12% sunlight, respectively. [file 12864_2020_7266_MOESM2_ESM.docx]

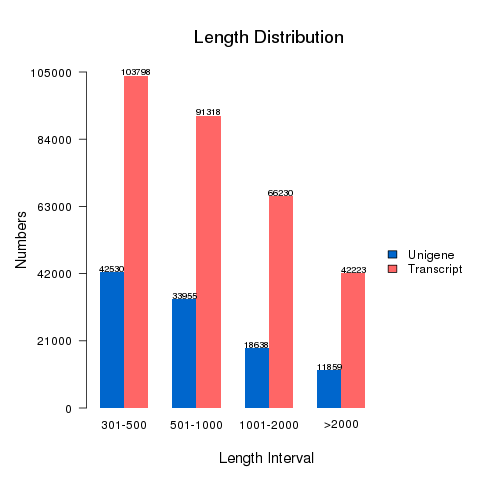


**Supplementary Figure 1.** Overview of transcriptome assembly data showing the size distribution of transcripts.


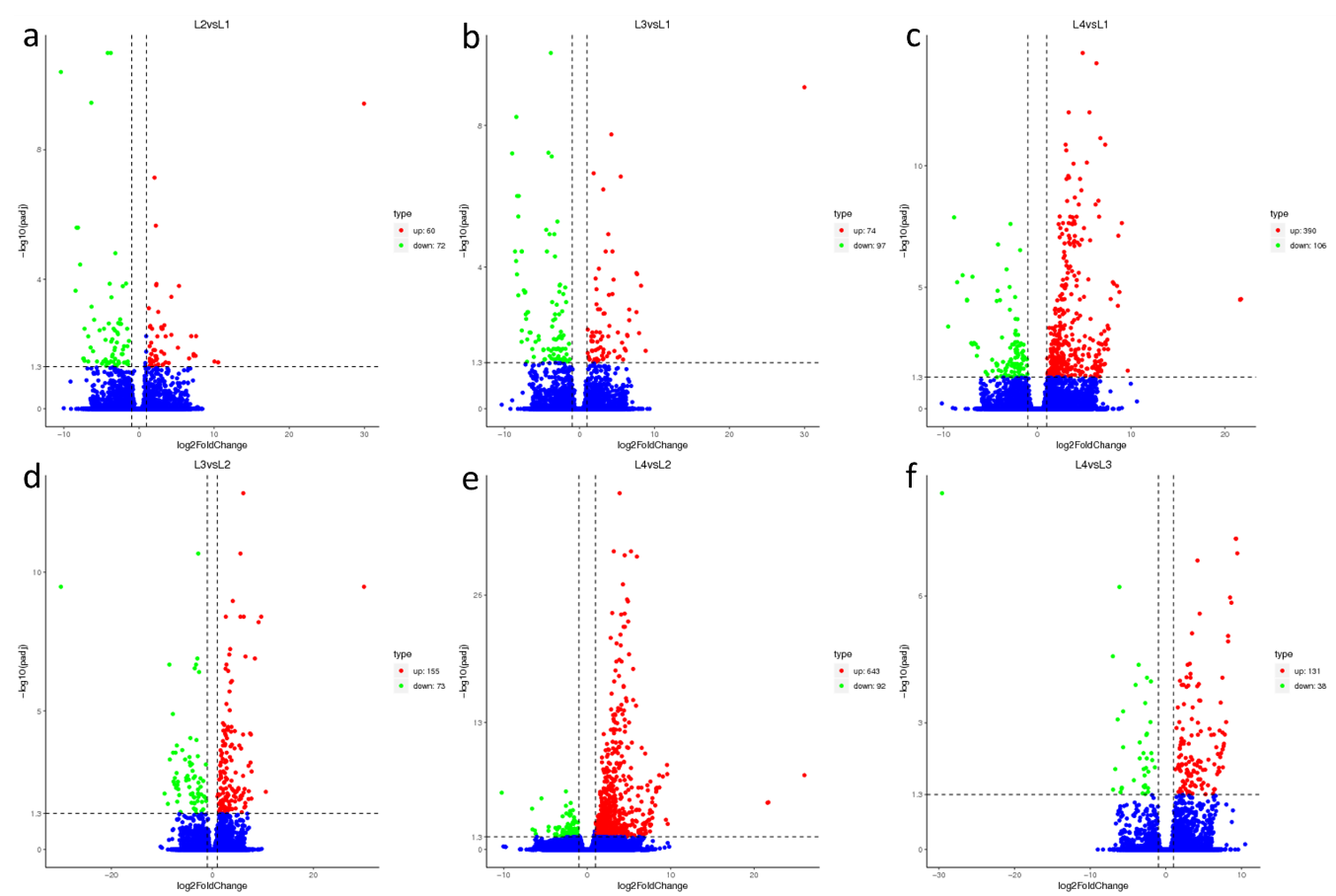


**Supplementary Figure 2.** Volcano plots of DEGs between treatments a) I vs II b) I vs III, c) I vs IV, d) II vs III, e) II vs IV, and f) III vs IV.


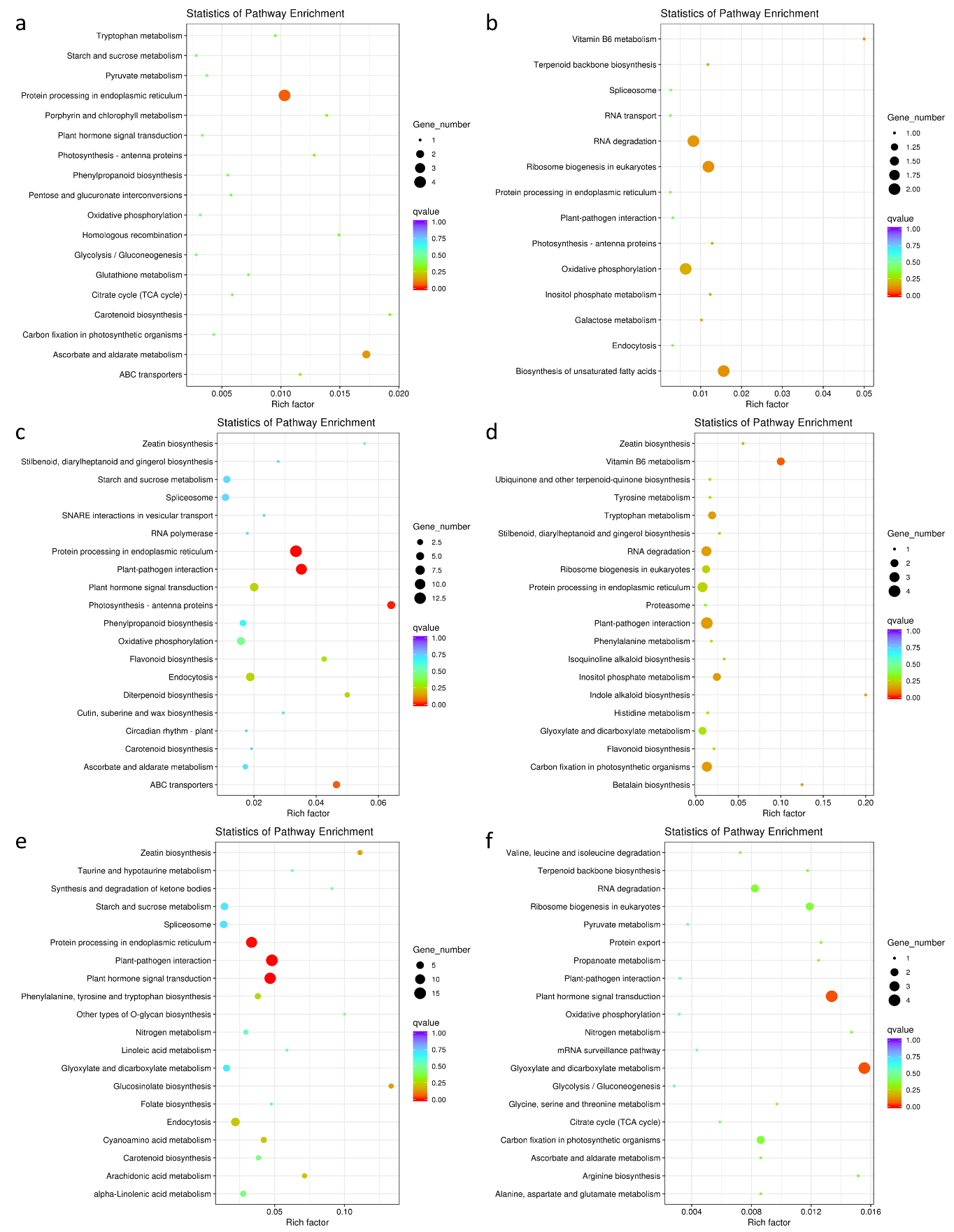


**Supplementary Figure 3.** KEGG enrichment analysis of DEGs between treatments a) I vs II b) I vs III, c) I vs IV, d) II vs III, e) II vs IV, and f) III vs IV.
